# New Prospects in Neutering Male Animals Using Magnetic Nanoparticle Hyperthermia

**DOI:** 10.3390/pharmaceutics13091465

**Published:** 2021-09-14

**Authors:** José Luiz P. R. Jivago, Juliana Lis Mendes Brito, Gustavo Capistrano, Marcus Vinícius-Araújo, Ediron Lima Verde, Andris Figueiroa Bakuzis, Paulo E. N. Souza, Ricardo Bentes Azevedo, Carolina Madeira Lucci

**Affiliations:** 1 Laboratory of Animal Reproduction, Department of Physiological Sciences, Institute of Biological Sciences, Campus Universitário Darcy Ribeiro, Brasilia 70910-900, DF, Brazil; jivago@unb.br (J.L.P.R.J.); juliana.lis@unb.br (J.L.M.B.); 2 Institute of Physics and CNanoMed, Federal University of Goiás, Goiania 74884-092, GO, Brazil; gustavokgustavo.capistrano@ifmt.edu.br (G.C.); mvinicius_1@discente.ufg.br (M.V.-A.); bakuzis@ufg.br (A.F.B.); 3 Instituto de Ciências Exatas e da Terra, Universidade Federal de Mato Grosso, Pontal do Araguaia 78060-900, MT, Brazil; ediron@ufmt.br; 4 Laboratory of Electron Paramagnetic Resonance, Institute of Physics, University of Brasilia, Brasilia 70910-900, DF, Brazil; psouza@unb.br; 5 Department of Genetics and Morphology, Institute of Biological Sciences, Campus Universitário Darcy Ribeiro, Brasilia 70910-900, DF, Brazil; razevedo@unb.br

**Keywords:** infertility, testicles, spermatogenesis, magnetohyperthermia, nanocontraception

## Abstract

Controlling populations of free-roaming dogs and cats poses a huge challenge worldwide. Non-surgical neutering strategies for male animals have been long pursued, but the implementation of the procedures developed has remained limited to date. As submitting the testes to high temperatures impairs spermatogenesis, the present study investigated localized application of magnetic nanoparticle hyperthermia (MNH) to the testicles as a potential non-surgical sterilization method for animals. An intratesticular injection of a magnetic fluid composed of manganese-ferrite nanoparticles functionalized with citrate was administered followed by testicle exposure to an alternate magnetic field to generate localized heat. Testicular MNH was highly effective, causing progressive seminiferous tubule degeneration followed by substitution of the parenchyma with stromal tissue and gonadal atrophy, suggesting an irreversible process with few side effects to general animal health.

## 1. Introduction

In recent decades, nanotechnology has been substantially introduced into biomedical applications, including reproductive biology and medicine. Most studies focused on improving the efficacy and precision of diagnostics and treatments for reproductive cancers and non-cancer conditions, while others investigated the use of nanomaterials as instruments in assisted reproduction and reproductive biology (see in [1] for a review). More recently, nanotechnology has been explored as a tool to develop male contraceptive methods [2,3,4,5,6], especially for animals.

Controlling populations of free-roaming animals, particularly dogs and cats, is a huge challenge worldwide. Such animals pose public health [7] risks as they may transmit diseases to domestic animals and humans [8,9,10]. They are also troublesome in urban environments as they spread domestic waste, cause traffic accidents, and attack people, not to mention their potential threat to disrupt the ecological balance by preying on wild animals. Culling cats and dogs is no longer deemed acceptable and has proven ineffective in controlling population growth. Neutering, on the other hand, is a more humane and effective alternative to control stray animal populations [11]. The so-called Trap-Neuter-Return (TNR) is a recent approach to control both stray and feral animal populations in the long term by creating groups of sterile animals, slowing the reproductive rate, and eventually decreasing the number of stray animals [7,12,13].

Although surgical castration is the technique of choice to neuter male animals, it presents complications, including risk of infection and the need for postoperative care [14,15,16,17]. Therefore, non-surgical neutering alternatives for male animals have been researched for a long time. Several studies investigated the effects of intratesticular or intraepididimal injections of sclerosing agents, such as zinc gluconate [18,19,20,21,22,23], calcium chloride [24,25], hypertonic saline [26], formalin, chlorhexidine gluconate, and zinc arginine [27,28,29]. However, these methods had limited use due to several undesirable side effects [18,21], and mainly because the resulting infertility was never proven to be permanent.

Alternative strategies to chemical sclerosing agents have also been investigated. It is well known that the testes are sensitive to temperature fluctuations, and that testicular hyperthermia causes structural and functional changes in the testes, which in turn impairs spermatogenesis [30,31]. Strategies exploring the heat sensitivity of testicular cells have been studied since the 1970s in search of a contraceptive method. An earlier study investigated several heating methods, including immersion in hot water, infrared light incidence, microwave, and ultrasound, together with different target temperatures (39, 40, 60, and 65 °C) [32]. More recent studies focused on using a water bath or ultrasound as approaches for thermal male contraception in rats [33,34,35,36] and dogs [37,38]. Although these methods are noninvasive and cause no, or only few, side effects, the induced infertility is reversible [34,36]. More importantly, the aforementioned techniques demand several applications and prolonged treatment which is undesirable for large-scale neutering method to be applied to unowned animals. Therefore, an optimized system capable of inducing testicular hyperthermia with a single procedure to achieve irreversible infertility is still needed.

Hyperthermia may be enhanced using magnetic nanoparticles (MNPs) as in the so-called magnetic nanoparticle hyperthermia (MNH). This technique exploits the unique physical properties of MNPs to increase the temperature in target tissues and/or organs. MNPs convert magnetic energy into heat [39], which only occurs when both the MNPs and an external AC magnetic field of adequate frequency and field amplitude are present. Therefore, this technique allows controlled and adjustable heating of a specific well-delimited region for a precise duration. Nowadays, MNH is under investigation as a therapeutic procedure to treat different types of tumors, with promising outcomes (for reviews see in [40,41]), and has been approved for clinical use to treat brain tumors [40].

Considering the high effectiveness of this method in producing uniform localized heat, we investigated testicular MNH as a potential animal sterilization method. The study evaluated the effect of the proposed treatment on the reproductive parameters of Wistar rats during spermatogenesis.

## 2. Materials and Methods

### 2.1. Magnetic Nanoparticles, Animals and Experimental Design

A magnetic fluid (MF) composed of manganese ferrite-based nanoparticles surface-coated with citrate (MnFe_2_O_4_-citrate) was used. The MnFe_2_O_4_-citrate nanoparticles were synthesized as reported by Branquinho et al. [42]. The magnetic nanoparticles were characterized by several techniques. Transmission electron microscopy (TEM) micrographs were obtained using a JEOL JEM 2100 (Jeol, Tokyo, Japan). Dynamic light scattering used Malvern Zetasizer Nano S equipped with a 633 nm He-Ne laser operating at an angle of 173 degrees. The concentration of nanoparticles was obtained by magnetic characterization using a vibrating sample magnetometer (model EV9, ADE Magnetics). In vitro magnetic hyperthermia measurements used the magneTherm 1.5 AC (nanoTherics, Warrington, UK) operating at 330 kHz. Temperature monitoring was performed using a LUXTRON 3300 m optical probe thermometer (LumaSense Technologies, Denver, CO, USA). In vitro specific loss power (SLP) estimation was obtained using the experimental initial MNH slope heating rate [42,43]. The in vivo magnetic nanoparticle hyperthermia experimental setup consisted of an Ambrell system model EasyHeat-LI (Ameritherm, Inc., Scottsville, NY, USA) operating at 301 kHz with a Helmholtz-like (2+2)-turn coil configuration that is cooled with a closed-loop circulating water system (maintained at 20 °C). The experimental setup also used an infrared thermal camera (model SC 620, FLIR, Wilsonville, OR, EUA). The AC field amplitude was determined in the region of interest using an AC field probe (AMF Lifesystems, Rochester, MI, USA). Experimental details can be found in [44].

Twenty-two 6–7-week-old male Wistar rats with a mean weight of 244.1 ± 31.6 g (ranging from 200 to 300 g) were maintained in groups of 4–5 animals in polyurethane cages at 25 °C, with a 12/12 h light/dark cycle, and ad libitum access to tap water and commercial food (Nutrina^®^). All animal experiments were approved by the Ethics Committee on Animal Use of the University of Brasilia (Protocol Number: 138067/2012).

**Figure 1:**
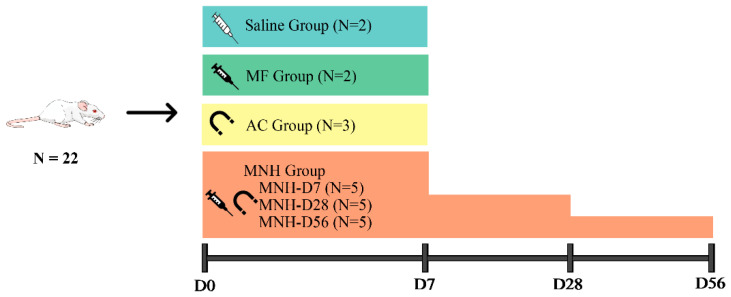
Schematic drawing of the experimental design. 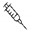 Saline Group, animals received an intratesticular injection of sterile saline solution. 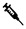 MF Group, animals received an intratesticular injection of the magnetic fluid. 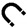 AC Group, animals were exposed to the external AC magnetic field only. 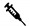 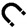 MNH Group, animals received an intratesticular injection of the magnetic fluid and were exposed to the external AC magnetic field. Animals were divided into 3 subgroups and evaluated: 7 (MNH-D7 Group), 28 (MNH-D28 Group), and 56 (MNH-D56 Group) days after the magnetic nanoparticle hyperthermia treatment.

The animals were divided into 4 groups according to the treatment received: (1) Saline Group (N = 2)—animals received an intratesticular injection of sterile saline solution; (2) MF Group (N = 2)—animals received an intratesticular injection of the magnetic fluid; (3) AC Group (N = 3)—animals were exposed to the external AC magnetic field only; and (4) MNH Group (N = 15)—animals received an intratesticular injection of the magnetic fluid and were subsequently exposed to the external AC magnetic field to produce testicular MNH. The MNH Group animals (N = 15) were divided into 3 subgroups (N = 5 per subgroup), to be evaluated: 7 (MNH-D7 Group), 28 (MNH-D28 Group), and 56 (MNH-D56 Group) days after the treatment (Figure 1).

### 2.2. Experimental Procedures

Prior to any experimental procedure, the animals underwent anesthesia with ketamine (90 mg/kg) and xylazine (10 mg/kg), intraperitoneally. The MF and MNH group animals received an intratesticular injection (150 µL) of the magnetic fluid in each testicle, applied equally at 3 different points (top, middle, and bottom) using a 32 G needle. Injected volume was calculated in a preliminary experiment to not excessively increase the organ’s pressure, and no fluid reflux was observed during the experiment. MNH Group animals were then positioned to expose their testicles to an alternating magnetic field operated at 300 kHz, with an average field amplitude of 240 Oe at the testes region. The protocol was similar to other studies conducted by the group [43,44]. Temperature was monitored using 3 fiber-optic temperature sensors: 1 sensor positioned on the surface of each testicle and the other inserted into the animal’s rectum to measure rectal temperature. The mean temperature at the surface of the testicles was also monitored using an infrared thermal camera following the protocol of Rodrigues et al. [43]. MNH group animals had the testicular temperature monitored for the entire time, and treatment was maintained for 15–20 min. AC group animals were anesthetized and exposed to the magnetic field for 15 min under the same conditions as the MNH group animals without receiving any intratesticular injection. Saline group animals received an intratesticular injection (150 µL) of sterile saline solution in the same manner.

After treatment, all animals received a single dose of analgesic and anti-inflammatory (Banamine—1.1 mg/kg SC). All animals were observed daily for behavior, change(s) in general appearance, signs of pain (according to grimace scale [45] and body condition [46]), food consumption, and weight until the end of the experiment.

The MNH-D7, MNH-D28, and MNH-D56 group animals were euthanized at 7, 28, and 56 days after the treatment, respectively. The animals in the other groups were euthanized 7 days after the treatment (Figure 1). The euthanasia was performed by anesthetic overdose (ketamine-xylazine) and cardiac puncture. The liver, kidneys, lungs, spleen, and testicles were harvested from all animals. Both testicles were measured (length and width), weighed, and processed for histopathological analysis. The other organs were weighed, with one sample fixed for histopathological analysis, and another frozen for the posterior quantification of MNPs by Ferromagnetic Resonance (FMR) [47].

Testicular volume was determined using testicular length and width, according to Louvandini et al. [48]. The absolute weight of each organ (liver, kidneys, lungs, spleen, and both testicles) was transformed into relative weight using the formula:

(1) $$\frac{organ\;weight}{body\;weight}\times \;100$$

### 2.3. Histological Processing

Organs were fixed in Bouin’s solution (testicles) or 10% formaldehyde (liver, kidneys, lungs, spleen) and processed for classical histology. Sample sections (5 µm thick) from each organ were stained with hematoxylin and eosin, and evaluated under a light microscope (Nikon Eclipse Ci-S, Tokyo, Japan).

### 2.4. MNP Detection and Quantification by Ferromagnetic Resonance (FMR)

To quantify the MNPs in each organ, the liver, kidneys, lungs, and spleen of all animals were individually macerated and homogenized in distilled water with an ultra-turrax^®^ (IKA^®^ Werke Staufen, Königswinter, Germany), and the homogenate lyophilized (L101, Liotop, São Carlos, SP, Brazil). Powdered samples were placed in capillary glass tubes, weighed and sealed. Samples were analyzed using an X-band in a Bruker spectrometer (Bruker EMX plus, Bremen, Germany) with a high-sensitivity cavity (Bruker ER 4119HS, Bremen, Germany). The spectra were collected at room temperature, 10 G modulation, 2 mW microwave power, and one scan. A calibration curve was constructed to determine the MNP concentration in the different organs using diluted samples of known MNP concentrations: 0.375 µg/mL, 0.75 µg/mL, 1.5 µg/mL, and 2.94 µg/mL.

The signaling detected by FMR is exhibited as a graph with 2 peaks, the amplitude measurement (peak to peak) acquired divided by the sample mass corresponds to the MNP concentration in each sample. The results obtained were represented in arbitrary units (A.U.) of nanoparticles per gram of dried tissue.

### 2.5. Statistical Analysis

All of the data were tested for normality using the Shapiro–Wilk test. Data were compared among groups by the ANOVA and Tukey tests using the GraphPad Prism 8.0.2 software (GraphPad Software, Inc., San Diego, CA, USA), with 5% considered as the minimum level of significance.

## 3. Results

### 3.1. Magnetic Nanoparticle Hyperthermia

**Figure 2:**
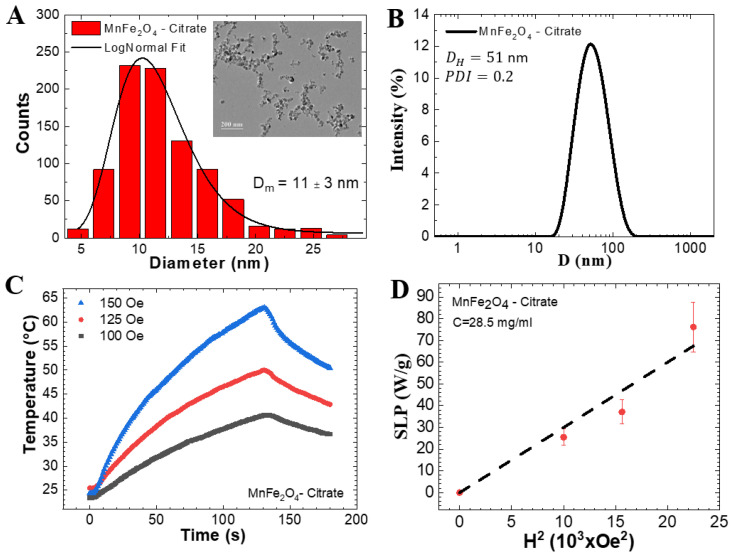
(**A**) Particle size distribution obtained from analysis of TEM pictures. Inset shows a representative TEM picture. (**B**) Dynamic light scattering data of the Mn-ferrite based colloid. (**C**) In vitro MNH temperature profile of a magnetic sample in three magnetic field amplitudes for a frequency of 333 kHz. (**D**) Specific loss power (SLP) as function of the square of the magnetic field. Data obtained using a particle concentration of 28.5 mg/mL.

The analysis of TEM pictures revealed spherical nanoparticles with a lognormal size distribution. The mean diameter was 11 nm with a standard deviation of 3 nm (see Figure 2A). In Figure 2B, the dynamic light scattering data revealed the colloidal hydrodynamic size distribution (mean diameter of 51.2 nm and PDI of 0.20). The in vitro MNH experiments used a particle concentration of 28.5 mg MnFe_2_O_4_ NPs per mL. Figure 2C shows the magnetic response of the magnetic colloid at different field amplitudes (100, 125, and 150 Oe) for a frequency of 333 kHz. The magnetic heating efficiency, also known as SLP, is shown in Figure 2D as function of the square of the field amplitude. The dash line is the best fit of the data using the linear response theory that predicts a linear dependence with H^2^. From this analysis we estimated the in vivo SLP, under the treatment’s magnetic field conditions (240 Oe), to be ~170 W/g.

**Figure 3:**
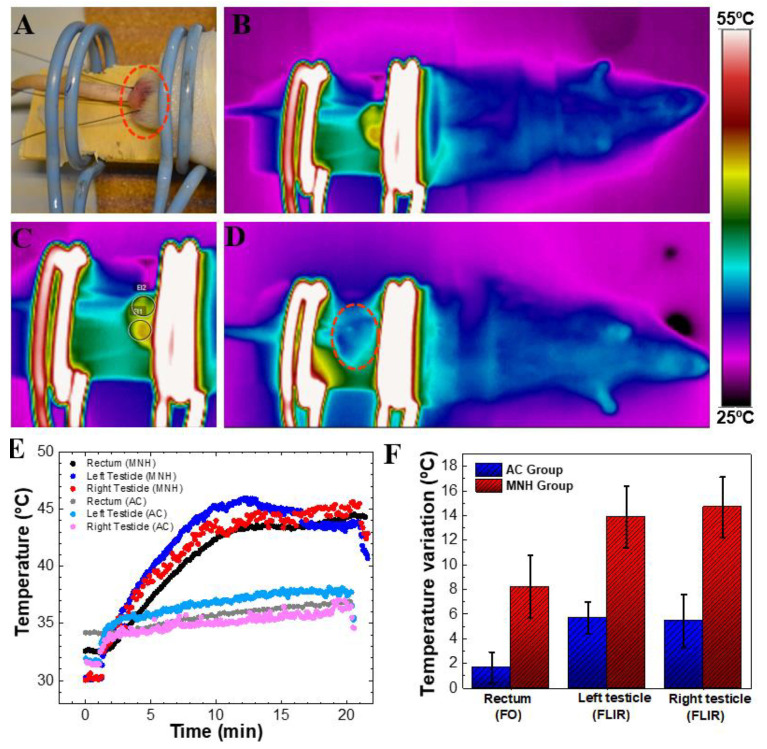
(**A**) Positioning of the animal’s testicles on the magnetic field coil. Note: fiber-optic probes to measure testicular and rectal temperatures. (**B**) Thermographic image of an animal during the procedure of magnetic nanoparticle hyperthermia when the average temperature of the testicles was 45 °C. It is possible to visualize that the only heated part of the body is the testicular area. (**C**) Photograph of the testicular region of the same (**B**) MNH group animal. (**D**) Thermographic image of an AC group animal after 15 min of magnetic field exposure. Note that no heat generation was observed in the testicular region. (**E**) Temperature data of both animals, MNH group and AC group, during treatment. Rectal temperature was obtained using a fiber-optic thermometer, while the left and right testicle temperatures correspond to an average value obtained using the thermal camera. (**F**) Temperature increase in AC and MNH groups considering all animals investigated (mean ± SD).

Figure 3 presents typical thermographic images of animals submitted to AC magnetic fields for 20 min. Figure 3D shows an AC group animal (that did not receive the MF injection), while Figure 3B shows an MNH Group animal. A temperature increase was observed in the MNH Group (peaking at ~45 °C), while no significant heat generation was observed in animals not injected with MNPs (AC Group). As expected, heat generation was restricted to the region injected with MNPs (see Figure 3C), i.e.**,** the testicles. Figure 3E shows the rectal and testicular (left and right) temperature profiles of both animals during the treatment. Rectal temperature was obtained with a fiber-optic temperature sensor inserted into the rectum, while the left and right testicular temperatures are mean values obtained using thermal camera analysis of a large region of interest within the testicles. One can observe that the rectal temperature is elevated and close to the testicle temperature data. Rectal temperature variation is high due to heat transfer from the testicles that are in close proximity to the rectum in the experimental configuration, which does not result in whole-body blood temperature increase, as can be observed by the thermal camara image (Figure 3B). Note that no animal died during the treatment, and we found no evidence of a surface temperature increase on any other part of the animal’s body. The AC Group showed a slight increase in temperature, achieving around 37 °C for this animal, while the MNH Group animal temperatures were close to 45 °C. The mean temperature increase during treatment for all animals in each group is shown in Figure 3F. In the left (right) testicle, we observed a temperature increase of 13.9 ± 2.49 °C (14.7 ± 2.45 °C) for the MNH Group, while for the AC Group the mean value increased to 5.68 ± 1.30 °C (5.45 ± 2.16 °C). This temperature rise was higher than observed in the rectum (1.66 ± 1.24 °C), suggesting that there was a slight temperature increase for the AC Group associated with free current loss. Once the hyperthermia treatment was completed, testicular temperature returned to basal within 4–5 min.

### 3.2. Clinical Observations

There were no complications during the MNH procedure in any of the treated animals. One animal of the MNH group died 3 days after the treatment. Although the autopsy did not indicate that the cause of the death was related to the treatment, this may not be discarded. All animals experienced typical weight gain associated with aging during the experiment, with no significant difference between groups (*p* > 0.05). Only a few animals presented some weight loss in the first 24 h after the treatment, which was not specific to the treatment group.

**Figure 4:**
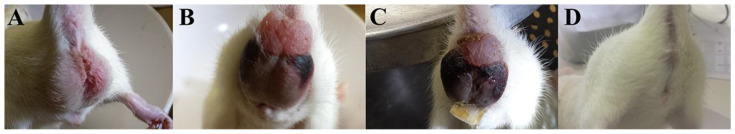
Macroscopic aspect of the scrotum of Wistar rats from (**A**) AC group (testicles exposed to the external AC magnetic field only) 7 days post-treatment, (**B**) MNH group (testicles injected with magnetic fluid and exposed to the external AC magnetic field) 4 days post-treatment, (**C**) MNH Group 15 days post-treatment, and (**D**) MNH Group 56 days post-treatment.

None of the animals showed any change in general appearance after the treatment. The animals in the Saline and AC groups presented a scrotum area with typical characteristics and color, without lesions of any kind (Figure 4A). The MF group animals presented testicles with a darkened aspect (observed through the skin) relating to the magnetic fluid injected.

All animals subjected to testicular MNH showed discrete swelling of the testicles and thinner scrotum skin for the first 7 days after treatment. These animals exhibited an abnormal behavior: constant licking of the scrotum. Skin lesions were observed in the cranial region of the scrotum in 57% (8/14) of the animals (Figure 4B). Despite this, the animals did not show signs of pain according to grimace scale [45] or body condition [46]. This inflammatory process gradually regressed and completely disappeared within 15 days (Figure 4C). In some animals, complete atrophy of the testicles and scrotum was observed 56 days after the treatment (Figure 4D).

### 3.3. Macroscopic Testicular Analysis

During the necropsy, the testicles of MNH-D7 group animals were dark and adhered to the scrotal skin. Animals from the MNH-D28 group presented testicular atrophy, and absence of the left testicle was observed in one animal. In animals from the MNH-D56 group, 3 testicles had disappeared (30% loss of testes in group MNH-D56-3/10 testicles), while the remaining testicles were atrophied and adhered to the scrotal wall. No macroscopic changes in the testicles were observed in animals of the Saline and AC groups, while the MF group animals only exhibited darkening of the testicles.

**Figure 5:**
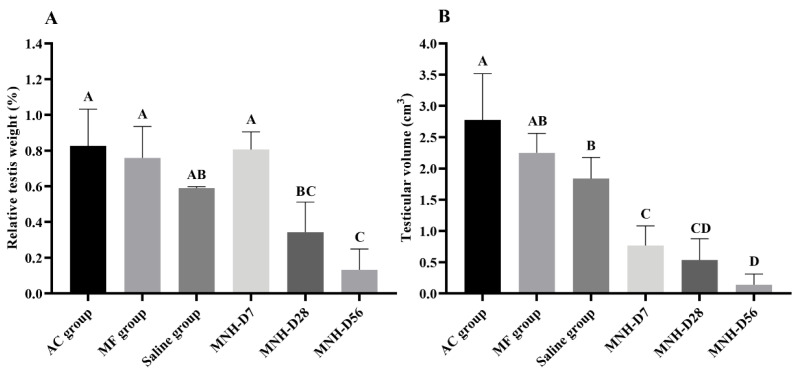
(**A**) Mean (±SD) relative testis weight (%) and (**B**) Mean (±SD) testicular volume (cm^3^) for each experimental group on the day of euthanasia. (ABC—different letters indicate significant difference, *p* < 0.05.) AC Group animal testicles were exposed to the external AC magnetic field only. MF Group animals received an intratesticular injection of the magnetic fluid only. Saline Group animals received an intratesticular injection of sterile saline solution. MNH Group animals received an intratesticular injection of the magnetic fluid and had their testicles exposed to the external AC magnetic field.

A significant decrease (*p* < 0.05) in relative testis weight was observed in the MNH-D56 and MNH-D28 groups compared to the MNH-D7, AC, MF, and Saline groups (Figure 5A). A significant decrease (*p* < 0.05) was also noticed in testicular volume in MNH-D7, MNH-D28, and MNH-D56 compared to the AC, MF, and Saline groups (Figure 5B).

### 3.4. Testicular Histopathology

**Figure 6:**
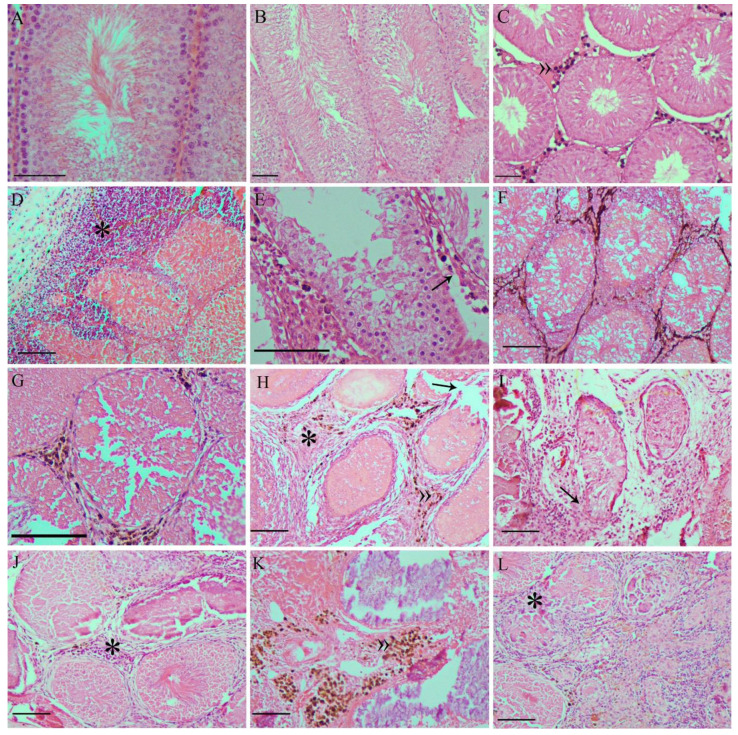
Representative photomicrographs of testicles from the: Saline (**A**), AC (**B**), and MF (**C**) groups, presenting normal seminiferous tubules and all germline cell types. In the MNH-D7 group (**D–F**), the seminiferous tubules presented damage to the germinal epithelium and rupture of the basement membrane was observed (**D**), in addition to blood leaking into the tubular lumen and inflammatory infiltrate (*) in the interstitial tissue (**E**). It was possible to identify nanoparticle agglomerates in the interstitium (**F**). In the MNH-D28 group (**G**–**I**), seminiferous tubules showed coagulative necrosis (**G**), the interstitial connective tissue was thickened (H), and the typical tubular structure was lost (**I**). Nanoparticle agglomerates are still visible in the interstitium (**G**,**H**). In the MNH-D56 group (**J**–**L**), seminiferous tubules lost definition (**J**,**K**) and began to be replaced by connective tissue (**L**), the nanoparticle agglomerates are still visible (K). »: nanoparticle agglomerates; black arrows: seminiferous tubule basement membrane rupture; *: inflammatory infiltrate. Bars = 10 µm.

All germline cells (spermatogonia, spermatocytes, spermatids, and spermatozoa) were observed inside the seminiferous tubules in animals of the Saline (Figure 6A) and AC (Figure 6B) groups, with tubular and stromal tissue structures intact. The same morphological characteristics were observed in the MF group, and it was also possible to identify agglomerates of nanoparticles in the interstitial tissue (Figure 6C).

MNH-D7 group animal testicles (Figure 6D–F) presented seminiferous tubules with a completely uncharacterized seminiferous epithelium. Spermatogonia were the only cells with normal morphology, while other germinative line cells, when present, had a vacuolized cytoplasm (Figure 6D). Some tubules presented blood leakage into the tubular compartment (coagulative necrosis), and inflammatory infiltrates were observed in the interstitial tissue (Figure 6E). In addition, aggregated nanoparticles were also visualized in the interstitial tissue (Figure 6F).

MNH-D28 group (Figure 6G–I) animal testicles possessed the most seminiferous tubules with an atypical structure. No germline cells were identified. The only cells inside the seminiferous tubules were Sertoli cells, albeit in a low number. Most tubules showed signs of coagulative necrosis (Figure 6G,H). The interstitial tissue was thicker, with more connective tissue (Figure 6H), which appears to be invading the lumen of the tubules (Figure 6I). Rupture of some seminiferous tubules could be seen (Figure 6H,I), together with nanoparticle agglomerates (Figure 6G,H).

Histopathological analysis of MNH-D56 group animal testicles (Figure 6J–L) showed an evolution of those presented by the MNH-D28 group. The seminiferous epithelium completely lost its definition and began to be replaced by connective tissue. In some animals, this exchange appeared to be complete (Figure 6L). Minor inflammatory infiltrates (Figure 6J), and nanoparticle agglomerates were still visible (Figure 6K).

### 3.5. Analysis of Vital Organs and MNP Quantification

The liver, spleen, lungs, and kidneys showed normal macroscopic appearance in all animals of all groups. No statistical difference (*p* > 0.05) was observed among groups regarding the relative weight of these organs (Table 1).

The FMR signals for all organs of animals that did not receive MF injections (i.e., Saline and AC groups) were insignificant (less than 110 a.u.). In all animals that received MF injections, the liver was the organ that showed the highest signaling, significantly more (*p* < 0.05) than the other organs, and significantly higher (*p* < 0.05) 7 days after the treatment (5427 a.u.) when compared to Day 28 (1376 a.u.) and Day 56 (1735 a.u.) post-treatment. The detection of MNPs in the spleen did not significantly (*p* > 0.05) change with time, with mean amounts of 1014, 240, and 222 a.u. on Days 7, 28, and 56 after the treatment, respectively. The amount of MNPs found in the kidneys (less than 65 a.u.) and lungs (less than 150 a.u.) was considered insignificant at all evaluated timepoints.

**Figure 7:**
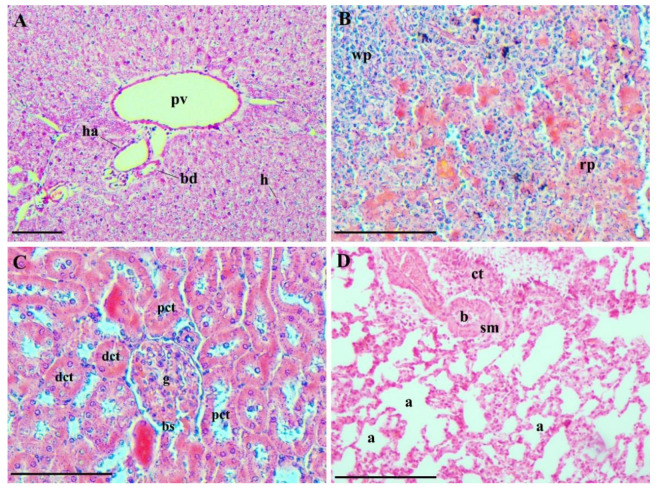
Representative photomicrographs of the evaluated vital organs: (**A**) liver (MNH-D28 group)—elements of the hepatic triad, branches of the portal vein (pv), a branch of the hepatic artery (ha), and a small bile duct (bd) are shown. Hepatocytes (h) are arranged in plaques radiating from the central venule regions and extend to the portal areas; (**B**) spleen (MNH-D7 group)—red and white pulp are distinguishable. The red pulp (rp) has several splenic cords, among which the sinusoids are located, and the white pulp (wp) is made up of lymphatic tissue, predominantly lymphocytes; (**C**) kidneys (MNH-D56 group)—note the glomerular capillaries (g) of a renal corpuscle, Bowman’s space (bs), proximal (pct) and distal (dct) convoluted tubules with normal appearance; and (**D**) lungs (MNH-D56 group)—the lungs of all animals were similar, and it was possible to observe pulmonary alveoli (a), alveolar sacs, bronchioles (b), pulmonary blood vessels, connective tissue (ct) and smooth muscle (sm). Bars = 10 µm.

Despite the detection of MNPs in the vital organs, histopathological analysis of the liver, spleen, lungs, and kidneys showed a normal overall appearance for the animals in all of the experimental groups (Figure 7).

## 4. Discussion

The present study investigated magnetic nanoparticle hyperthermia applied locally to the testicles as a possible animal neutering method. The results showed that this approach was effective, causing progressive degeneration of seminiferous tubules, followed by substitution of the parenchyma by stromal tissue and gonadal atrophy, suggesting that the process is irreversible.

The harmful effects of heat on spermatogenesis are well documented [49]. When testicles do not descend to the scrotal sac and remain within the abdomen—a condition termed cryptorchidism, the animal is sterile, although testosterone production is not affected [50]. Transient heating of the testicles (40–45 °C), due to high ambient temperatures or testicle insulation, for example, also causes germ cell degeneration and impairs spermatozoa production [34]. In the present work, animal testicles were subjected to ~45 °C for 15 min in a single procedure of magnetic nanoparticle hyperthermia. This was sufficient to cause a rapid degenerative process of seminiferous tubules, observed as early as 7 days after the procedure. The damage was progressive, with disruption of tubule structure, extravasation of blood into the lumen (coagulative necrosis), and finally the replacement of germinal tissue with connective tissue.

Testicular hyperthermia has been previously investigated as a contraceptive method for both humans [32] and animals [37,38]. These studies used either immersion in hot water or ultrasound to heat the testicles and generally required multiple applications for days or weeks to induce reversible infertility [32,33,34,35,36,37,38,39]. However, while temporary effects are intended for humans, irreversible infertility is preferred for animals. The MNH strategy proved effective in a single procedure and caused irreversible effects. This may be due to the homogeneity of the heat generated. During MNH, the magnetic moment of the nanoparticles interacting with the alternating magnetic field generates heat. Unlike the outside to inside heating of a water bath, the heat generated in MNH spreads from inside the tissue to the outside. As the nanoparticles are well distributed in the testes and the magnetic field can easily penetrate the body, the heat generated within the organ is homogeneous. This is an advantage in comparison to water bath heating, ultrasound, and photothermal treatments. Although the use of ultrasound and immersion in hot water are less invasive treatments, MNH improved the effect of heat in gonadal tissue, enabling an optimized hyperthermia procedure in a single application.

Note that testicular MNH only increased the temperature of the target region. Although a slight increase in rectal temperature was observed when measured by the intrarectal fiber optical probe (observed in Figure 3E,F), the thermographic image of the animal showed that there was no rise in body temperature (visualized in Figure 3B). This could be due to the proximity of the rectum to the target area, which does not reflect the body temperature. Moreover, this slight localized increase in rectal temperature did not pose a threat to the body. This corroborates the clinical finding, where no animal died as a result of the heat convection mechanism. This differs from whole-body MNH in which nanoparticles may accumulate in the liver, with significant temperature increases in the liver and blood resulting in animal death [51].

Only a few nanotechnological strategies to cause infertility in male animals have been previously reported. The pioneering work showed that a hyperthermia treatment mediated by the testicular injection of methoxy-poly(ethylene glycol)-modified gold nanorods followed by near-infrared irradiation can induce temporary or permanent infertility in male mice, depending on the power of the NIR irradiation [52]. Subsequent studies proposed a procedure using the combined photothermal and photodynamic effects caused by intratesticular injection of single-layer WO_2.72_ nanosheets [2] or plasmonic copper sulfide nanocrystals (Cu_2_x_S NCs) [3] followed by NIR laser irradiation. The aforementioned treatments caused the destruction of testicular tissue, decreased testosterone plasma levels in mice within 14 days, and no recovery of reproductive functions until two months after the procedure. More recently, a single intratesticular injection of silver nanoparticles showed acute, but reversible, effects on spermatogenesis in rats [6]. All of these treatments showed no side effects on animal health over a short duration. However, as the light penetration of tissues employed in photothermal therapy is reduced, less homogeneous heat distribution may result within the testes, which could impact clinical results. This effect is pronounced in animals with large testes due to inhomogeneous heat distribution within the organ. On the other hand, MNH does not have this limitation since the magnetic field can easily penetrate the organ. Furthermore, iron-oxide nanoparticles have a relatively low cost, are widely used in clinics, and are easily synthesized on a large scale.

The primary degenerative process observed in the testicles submitted to MNH was coagulative necrosis. Coagulative necrosis is also observed when sclerosant agents are injected into the testicles [18,20,22,23], although none of the studies using this approach affirmed that this method induces permanent infertility. In the present study, however, the complete replacement of the parenchyma by stromal tissue strongly suggests that the damage is permanent. In fact, gonadal atrophy was observed in all MNH-treated animals, and testicle disappearance was witnessed in a few cases. Moreover, although testosterone levels were not monitored at this time, the extension of testicle fibrosis suggests that hormonal production was also impaired. Chemical castration by injecting sclerosant agents into the testicles causes several undesirable side effects, such as scrotal swelling, necrotizing dermatitis, ulceration, pain, and inflammation [18,21]. Regarding testicular MNH, the only side effects observed were weight loss during the first 24 h, a mild local inflammatory process causing discomfort (noticed by the constant licking of the area), and a skin lesion on the scrotum (observed for 8 of the 14 treated animals). The weight loss may simply be a consequence of the anesthesia, as the animals are less alert on the first day and do not have typical daily consumption. The inflammatory process is expected and may be addressed with a stronger anti-inflammatory treatment (in this experiment, we used a low dose of Banamine only). The skin lesion may be a consequence of the heating or constant licking of the area, as the scrotum skin is very thin and delicate. In treatments using ultrasound, skin lesions occurred in 20% of cases [53,54]. In the present study, the lesions healed entirely within 15 days. Furthermore, note that not all treated animals presented lesions and that no behavioral or growth alterations were observed in those which did. Nevertheless, the method must be improved to avoid undesirable effects.

Animals that received the MF injection only or those submitted to the magnetic field only did not exhibit any macroscopic or microscopic alterations in testicular tissue (apart from a dark aspect on testicles injected with MF). Moreover, no alterations were observed regarding the relative weight or histology of the liver, spleen, kidneys, or lungs of animals injected with the MF (submitted to MNH or not). Magnetic nanoparticles have been used for different biomedical applications, including hyperthermia, magnetically targeted delivery, and molecular magnetic resonance imaging diagnostics. In fact, contrast agents-based iron nanoparticles have been approved for human use by the US Food and Drug Administration in the USA. It is already known that when MNPs are injected intravenously they are distributed preferentially into liver, spleen, but depending on the animals’ models and the coating present in them, they can also be found in the lungs and kidney [47,55,56,57,58,59,60]. Although, MNPs can persist for months inside these organs, mainly in the liver and spleen, the great majority of the studies shows that they do not cause impairment of the cited organs, and in the long term, they are metabolized and excreted into the blood stream in various monoatomic iron forms, that can be incorporated into hemoglobin or into ferritin and transferrin [47,55,56,58,59,60,61,62].

In the present study, the magnetic signal of the nanoparticles was mainly observed in the liver 7 days after the injection. The second organ presenting a relatively high signal was the spleen. The signal was lower 28 and 56 days after the injection, in both the liver and the spleen, suggesting MNPs are eliminated from the body. Even so, the signal remained high in the liver of treated animals 56 days after the injection, and nanoparticle aggregates were still visible in the testicular tissue. Most iron nanoparticles accumulate in the liver and spleen [41], which is attributed to nanoparticle recognition by the reticuloendothelial system [63]. A previous study, using the same nanoparticles used in the present study, also showed a higher concentration in the liver and the spleen after intravenous injection [64]. However, it seems that the nanoparticle’s kinetics, when injected into the testicles, is different from that of intravenous administration. In the latter, there is a fast increase of nanoparticles in the liver, which are eliminated through hepatic clearance (feces) within the next few weeks [64]. Differently, in the administration form used in the present study (i.e., intratesticular injection), the nanoparticles remain in the testicles longer and slowly pass into the bloodstream, resulting in later accumulation in the liver. It has been described that iron-oxide nanoparticles are only excreted slowly in small amounts in urine and feces [65], and that the particle size has a strong influence in the elimination route [66]. In this work, nanoparticles in the range of 11 ± 3 nm were used, which accumulate in the liver [64] and are eliminated through hepatic clearance (feces) [66]. The accumulation of iron nanoparticles in tissues is a general concern because the body lacks a mechanism to eliminate excess iron and high levels of iron cause oxidative stress in tissues [67]. Nonetheless, iron-oxide nanoparticles continue to be actively investigated and used due to their potential for rapid heating of target tissues, high biocompatibility (once modified with a stabilizing layer), and minimal toxic effects [68]. In the present study, no clinical signs of iron toxicity (described as swollen snout and/or paws, labored respiration, and red crust around the nose [65]) or histopathological findings in vital organs were observed probably because the dose used (0.03–0.04 mg/Kg) was too low to induce systemic effects. However, it was sufficiently high to promote localized heating.

## 5. Conclusions

In conclusion, testicular magnetic hyperthermia mediated by MnFe_2_O_4_-citrate nanoparticles, as performed under the conditions described in this study, proved effective in neutering male rats in as short a time as 56 days with few side effects on animal general health. The procedure proposed here presents advantages over surgical castration as there is no need for a sterile room, instruments, or follow-up treatments, such as long-term antibiotic therapy and stitch removal. Moreover, this MNH procedure is better than chemical sterilization in that it induces a milder inflammatory response than sclerosing agents. However, a long-term follow-up of treated animals must be performed to evaluate any possible late side effects and prove the irreversibility of infertility.

## Figures and Tables

**Table 1:** Mean (± SD) relative weight (%) of organs on the day of euthanasia for the different experimental groups.

Organ	AC	MF	Saline	MNH-D7	MNH-D28	MNH-D56
Liver	3.56 ± 0.37	4.23 ± 0.13	4.36 ± 0.01	4.50 ± 0.74	3.94 ± 0.43	3.26 ± 0.16
Spleen	0.35 ± 0.02	0.63 ± 0.04	0.49 ± 0.08	0.57 ± 0.13	0.48 ± 0.08	0.35 ± 0.04
Lungs	0.65 ± 0.18	0.60 ± 0.01	0.63 ± 0.01	0.73 ± 0.21	0.52 ± 0.08	0.50 ± 0.06
Kidneys	0.46 ± 0.02	0.43 ± 0.01	0.41 ± 0.01	0.52 ± 0.19	0.43 ± 0.02	0.41 ± 0.04

AC Group animal testicles were exposed to the external AC magnetic field only. MF Group animals received an intratesticular injection of the magnetic fluid only. Saline Group animals received an intratesticular injection of sterile saline solution. MNH Group animals received an intratesticular injection of the magnetic fluid and had their testicles exposed to the external AC magnetic field.

## Data Availability

The data presented in this study are available on request from the corresponding author. The data are not publicly available due to confidentiality agreements.
